# Isolation methodology is essential to the evaluation of the extracellular vesicle component of the senescence‐associated secretory phenotype

**DOI:** 10.1002/jev2.12041

**Published:** 2021-02-18

**Authors:** Ryan Wallis, Natasa Josipovic, Hannah Mizen, Arturo Robles‐Tenorio, Eleanor J. Tyler, Argyris Papantonis, Cleo L. Bishop

**Affiliations:** ^1^ Blizard Institute of Cell and Molecular Science Barts and The London School of Medicine and Dentistry London UK; ^2^ Institute of Pathology University Medical Centre Göttingen Göttingen Germany

**Keywords:** ageing, exosomes, extracellular vesicles, paracrine senescence, senescence‐associated secretory phenotype, senescence

## Abstract

A hallmark of senescence is the acquisition of an enhanced secretome comprising inflammatory mediators and tissue remodelling agents – the senescence‐associated secretory phenotype (SASP). Through the SASP, senescent cells are hypothesised to contribute to both ageing and pathologies associated with age. Whilst soluble factors have been the most widely investigated components of the SASP, there is growing evidence that small extracellular vesicles (EVs) comprise functionally important constituents. Thus, dissecting the contribution of the soluble SASP from the vesicular component is crucial to elucidating the functional significance of senescent cell derived EVs. Here, we take advantage of a systematic proteomics based approach to determine that soluble SASP factors co‐isolate with EVs following differential ultracentrifugation (dUC). We present size‐exclusion chromatography (SEC) as a method for separation of the soluble and vesicular components of the senescent secretome and thus EV purification. Furthermore, we demonstrate that SEC EVs isolated from senescent cells contribute to non‐cell autonomous paracrine senescence. Therefore, this work emphasises the requirement for methodological rigor due to the propensity of SASP components to co‐isolate during dUC and provides a framework for future investigations of the vesicular component of the SASP.

## 1 INTRODUCTION

Senescence is a permanent state of cell cycle arrest which occurs in cells exposed to a variety of potentially damaging stimuli including: telomere attrition; DNA‐damaging agents; irradiation; hydrogen peroxide (H_2_O_2_); and oncogene‐expression (Gorgoulis et al., 2019; Sharpless & Sherr, 2015). This allows senescence to act as a protective mechanism, preventing the malignant transformation of cells (Munoz‐Espin & Serrano, 2014). However, senescent cells also accumulate with age, and have been demonstrated to contribute to a range of age‐related diseases (e.g. Baar et al., 2017; Baker et al., 2016; Childs et al., 2016; Ogrodnik et al., 2017; Xu et al., 2018). These pathologies are driven by the acquisition of an enhanced secretome comprising inflammatory cytokines, chemokines, growth factors and proteases – the senescence‐associated secretory phenotype (SASP) (Coppé et al., 2008; Coppé et al., 2010). Through these factors, senescent cells have been demonstrated to propagate within the local microenvironment via the induction of senescence in neighbouring proliferating cells, in a process termed paracrine senescence (Acosta et al., 2013). This phenomenon is hypothesised to contribute to both the accumulation of senescent cells with age, and a low level, chronic state of inflammation which underpins a plethora of inflammatory age‐related pathologies (Coppé et al., 2010). The SASP is also a heterogeneous phenotype, varying in composition between cell types and senescence triggers, as well as dynamically changing as cells transition from proliferation to senescence (Basisty et al., 2000; Coppé et al., 2010; Hoare et al., 2016).

Adding to this complexity is recent evidence highlighting the role of extracellular vesicles (EVs) within the SASP (Takasugi, 2018). These small, lipid‐bilayer bound particles represent a heterogeneous set of mediators which vary in size, biogenesis and composition but are often defined in two major subclasses – exosomes and microvesicles (MVs) (Van Niel et al., 2018). These principally differ in their route of biogenesis with MVs budding directly from the plasma membrane and exosomes deriving from intraluminal vesicles within endosomal multivesicular bodies (Colombo et al., 2014). There are currently no universally accepted biomarkers with which to distinguish these populations, with size being the most widely used method of distinction. Exosomes are generally considered smaller, ranging from 30 to 150 nm in dimeter, whilst MVs have a broad size‐range of 50–1000 nm, although given overlapping size profiles and heterogeneity within these two populations, confident designation is difficult. To reflect this ambiguity, we use the recommended term ‘EVs’ throughout this manuscript (Witwer & Théry, 2019).

EV production has recently been demonstrated to increase in models of therapy‐induced (Kavanagh et al., 2017; Takasugi et al., 2017), H_2_O_2_‐induced (Terlecki‐Zaniewicz et al., 2018), replicative (Takasugi et al., 2017) and oncogene‐induced (Borghesan et al., 2019; Takasugi et al., 2017) senescence. Furthermore, EVs derived from senescent cells have recently been demonstrated to have functional roles, including paracrine senescence (Borghesan et al., 2019), enhanced cancer‐cell proliferation (Takasugi et al., 2017), apoptosis resistance (Terlecki‐Zaniewicz et al., 2018) and homeostatic DNA‐damage responses (Takahashi et al., 2017). These studies indicate that EVs are functionally important mediators within the SASP, giving them potential biological significance within both ageing and pathologies associated with age (Wallis, Mizen, & Bishop, 2020).

In order to elucidate these potential roles, EVs must be separated from the soluble secretome of the producing cells. However, many widely applied isolation techniques, including the popular differential ultracentrifugation (dUC) method, are unable to separate EVs from soluble proteins (and has been indicated to produce minimal soluble contamination of isolated EV preparations (Foers et al., 2018, Théry et al., 2018). Size‐exclusion chromatography (SEC) is an increasingly popular alternative or complimentary method for EV isolation. This study aims to investigate the potential for EV contamination with soluble factors when applying dUC as the sole method of isolation. We propose that co‐isolation of SASP components during dUC is an underappreciated issue within the field, complicating compositional and functional separation of the soluble and vesicular components of the senescent secretome. We demonstrate that SEC is efficient at minimising this issue, allowing elucidation of senescent cell derived EV composition by removing contaminating soluble SASP factors. Furthermore, SEC allows for comparison between the functional effects of both the soluble and vesicular SASP. We demonstrate that EVs isolated from oncogene‐induced senescent (OIS) IMR90 fibroblasts contribute to the paracrine signalling of the SASP in both proliferating IMR90 fibroblasts and MDA‐MB‐468 breast cancer cells. Therefore, this study highlights the limitations of dUC as a method of studying senescent cell derived EVs and suggests SEC as an alternative, more stringent technique.

## 2 MATERIALS AND METHODS

We have submitted all relevant data of our experiments to the EV‐TRACK knowledgebase (EV‐TRACK ID: EV200014) (Van Deun et al., 2017).

### 2.1 Cell culture and reagents

Unless otherwise stated, all reagents were purchased from Sigma, UK. MDA‐MB‐468 breast cancer cells were purchased from ATCC and maintained in Dulbecco's Modified Eagles Medium (DMEM; Life Technologies, UK) supplemented with 10% foetal bovine serum (FBS, Labtech.com, UK), 2 mM L‐glutamine (Life Technologies, UK) and 1 mM sodium pyruvate. *IMR90 ER:STOP* (vector) *or ER:RAS* (OIS) foetal lung fibroblasts were produced as described in (Hari et al., 2019) and were a kind gift provided by Juan Carlos Acosta (MRC Institute of Genetics & Molecular Medicine, Edinburgh). These were maintained in DMEM supplemented with 10% FBS and 2 mM L‐glutamine. Primary adult human mammary fibroblasts (HMFs) were kindly donated by Martha Stampfer (Lawrence Berkeley National Laboratory, Berkeley) and cultured in the same medium as IMR90s, with the addition of 10 μg/ml bovine pancreas insulin. All cells were maintained at 37°C/5% CO_2_, routinely tested for mycoplasma, and shown to be negative. Cells were grown in media without antibiotics apart from during EV treatments where penicillin‐streptomycin (50 units (U)/ml and 50 μg/ml final concentration, respectively) (Life Technologies, UK) was used.

### 2.2 Senescence induction

Vector and OIS IMR90 cells were seeded at 10,000 cells/cm^2^ and treated with 200 nM 4‐hydroxytamoxifen (4‐OHT) in DMEM with 10% FBS on 1 day post seeding. On day 4, media was then changed, and cells cultured in DMEM with 4‐OHT and 1% exosome‐depleted FBS (Gibco, UK) until day 8. At this point, media was collected, and cells were passaged into 96‐well plates. These were cultured for a further 5 days at which point immunofluorescence staining and high content analysis (HCA) of senescence markers was performed. This represents an optimised protocol, with alternative iterations utilising seeding densities of 2000 cells/cm^2^ and 4‐OHT doses of 100 nM to facilitate later time points. Details are specified in figure legends.

Replicative senescence in adult HMFs was induced through serial passaging of cells for over 200 days. Cells were designated as either early (passage 10–16; EP) or late (passage ≥26; LP) passage to indicate their number of cumulative population doublings. For senescence phenotyping by HCA, cells were seeded at 10,000 (EP) and 15,000 (LP) cells/cm^2^ and cultured for 5 days following by fixation and immunofluorescence staining. For EV isolation experiments, cells were seeded at 7500 (EP) and 15,000 (LP) cells/cm^2^ and cultured in media containing 10% exosome‐depleted FBS for 72 h between days 4 and 7 post seeding.

### 2.3 Immunofluorescence staining and high content analysis senescence phenotyping

Cells in 96‐well plates were washed with PBS and fixed using 3.7% paraformaldehyde (PFA) supplemented with 5% sucrose for 15 min at room temperature. Cells were washed with PBS and permeabilised using 0.1% Triton X‐100 for 15 min at room temperature. Cells were washed with PBS and blocked with PBS 0.25% (w/v) bovine serum albumin (PBS/BSA) for 30 min before incubation with primary antibody diluted in PBS/BSA overnight at 4°C. Cells were then washed with PBS/BSA for 30 min at room temperature and incubated with the appropriate Alexa Fluor‐546 conjugated secondary antibody (1:500, Invitrogen), 4’,6‐diamidino‐2 phenylindole (DAPI) (Sigma UK, D8417, 1:1000) and HCS Cell Mask Deep Red (Thermo‐Fisher UK, C10046, 1:50,000) for 2 h at room temperature. Cells were then washed with PBS/BSA for 30 min before three final PBS washes. Images were acquired using the IN Cell 2200 automated microscope (GE) and HCA was performed using the IN Cell Developer software v1.9.2 (GE). In order to characterise the induction of a senescence phenotype, a high‐content analysis based assessment of established senescence‐associated morphological alterations was employed (Hwang et al., 2009; Neurohr et al., 2019; Sadaie et al., 2015; Zhao & Darzynkiewicz, 2013). This lead to production of a morphological profile defined by the following measures: ‘Cell Number’, ‘Cell Area’, ‘Nuclear Area’, ‘Cytoplasmic/Nuclear Ratio’, ‘DAPI Density’, ‘Nuclear Form Factor’, ‘Cellular Protrusions’, ‘Cellular Form Factor ’, ‘Major Axis Length’, ‘Minor Axis Length’, ‘Cellular Elongation’. Z‐scores relative to the proliferating control were then calculated using the following equation to provide a means of data scaling: **Score =** mean value of three independent experiments for OIS experimental condition **–** mean value of three independent experiments for vector control condition/standard deviation (SD) of vector control condition. Z scores were then represented as heat maps, with maximum (+/‐ five Z‐scores) and minimum (+/‐ one Z‐scores) thresholds in order to demonstrate positive or negative modulation from the control condition. Importantly, this approach was validated in the OIS model via the use of complimentary canonical senescence markers: p21, p16, Ki67, IL‐8 and senescence‐associated heterochromatin foci (SAHFs).

### 2.4 Extracellular vesicle isolation

#### 2.4.1 Differential ultracentrifugation

Senescence induction procedures were performed according to the protocols described above and final mean cell numbers are indicated in figure legends. During conditioning, cells were switched to media containing exosome‐depleted FBS. This was demonstrated to contain undetectable levels of EVs and did not alter the proliferation of HMFs seeded at 10,000 cell/cm^2^, when treated for 48 h between day 4 and 6 post seeding (Figure S1). Conditioned media was then collected and centrifuged at 2,000 x *g* for 10 min at 4°C to remove dead cells, apoptotic bodies and cellular debris. The supernatant was then transferred to 50 ml polypropylene tubes (Nalgene UK, 3118‐0050) and centrifuged at 10,000 x *g* for 30 min at 4°C (Sorvall RC6+ High Speed Rotor: SS‐34, RPM: 9130, k‐factor: 3,598.4). The supernatant was then transferred to 30ml Oak Ridge polycarbonate tubes (Thermo Scientific, UK) and ultracentrifuged at 100,000 x *g* for 1 h 30 min at 4°C (Sorvall Discovery 100 SE Rotor: T‐865, RPM: 31,300, k‐factor: 223.1). The supernatant was discarded and the pellet resuspended in 250 μl PBS (Sigma, UK) which had been filtered through a 0.22 μm sterile filter (VGR UK, 514‐0061). These preparations were stored at ‐80°C.

### 2.5 Size‐exclusion chromatography

Differential centrifugation was carried out as above with the final resuspension volume adjusted to 500 μl. This was then loaded on to qEV original SEC columns (Izon Science, UK). Twenty sequential fractions of 500 μl were collected as per manufacturer's instructions. Characterisation was then performed by nanoparticle tracking analysis (NTA), microBCA and immunoblotting.

#### 2.5.1 Nanoparticle Tracking Analysis (NTA)

NTA was performed using NS300 (Malvern‐Panalytical, UK). EVs were diluted in 0.22 μm sterile filtered PBS to a volume of 1 ml. This dilution was appropriate to achieve a final particles per frame of between 15 and 80, as recommended by the manufacturer. However, where samples had a low particle concentration, a maximum dilution of 1:50 was used in order to facilitate other methods of analysis. Camera level 15 and detection threshold 3 in software version NTA 3.2 were used.

### 2.6 MicroBCA

EV preparation protein concentrations were determined via microBCA protein assay kit according to the manufacturer's instructions (Thermo‐Fisher, UK). Samples were read at an absorbance of 562 nm using a Synergy HT plate reader (Bio‐Tek, UK).

### 2.7 Immunoblotting

Cells were lysed in RIPA buffer supplemented with 4% protease cocktail inhibitor (Roche) and protein concentration was determined using the Bio‐Rad Protein Assay kit (Bio‐Rad). Lysates were re‐suspended in 6X Laemmli Sample Buffer (0.1 M Tris pH 6.8, 20% glycerol, 1% β‐mercaptoethanol, 1% sodium dodecyl sulphate (SDS), 0.01% bromophenol blue). EV samples were loaded according to either particle number (3e9 particles; ultracentrifugation only) or volume (25 ul; SEC) along with 6x Laemmli Sample Buffer but were not lysed. Gels were prepared and run using the Bio‐Rad Mini‐PROTEAN III system (Bio‐Rad, UK). Membranes were then blocked for 1 h in 0.05% (v/v) Tween‐20, 5% (w/v) Marvel semi‐skimmed milk in PBS (PBS‐T‐milk) at room temperature. The primary antibody was diluted in PBS‐T‐milk and incubated with the membrane overnight at 4°C. Primary antibodies used are listed in below. The membrane was then washed in PBS‐T for 3 × 5 min. Secondary antibodies were then prepared in PBS‐T‐milk and the membranes incubated for 1 h at room temperature. A further 3 × 5 min PBS‐T wash was carried out followed by Enhanced Chemiluminescence (ECL) (GE Healthcare, UK) and the membrane then exposed to a photographic film (Hyperfilm, GE Healthcare, UK).

### 2.8 Mass Spectrometry (MS) analysis

#### 2.8.1 Sample preparation

Purified EVs were resuspended in 1:1 ratio with 8 M Urea/50 mM TEAB buffer supplemented with 1x Protease Inhibitor Cocktail. Samples were sonicated using a Bioruptor for 10 min at 30/30s on/off cycles and centrifuged for 15 min at 20,000 x *g*. Supernatants were incubated with 5 mM DTT for 1h at 25°C, after which 40 mM chloroacetamide (Merck, Germany) was added and incubation was carried out for 30 min at 25°C in the dark. Samples were treated with 0.1 ug/μl of Lysyl Endopeptidase (Wako, Germany) at room temperature for 4 h and subsequently with 0.1 ug/μl of Trypsin (Serva, Germany) overnight. The next day, samples were acidified with 1% of formic acid (FA; Honeywell, Germany) and stage tip purification was performed. Stage tips were activated with through serial washes with 100% Methanol (VWR, Germany), 0.1% FA in 80% Acetonitrile (Merck, Germany) and 0.1% FA in water. After sample loading, stage tips were washed with 0.1% FA in water and 0.1% FA in 80% acetonitrile. Stage tips were air dried and stored at 4°C. Label‐free mass spectrometry was performed using a nanoHPLC coupled to a Thermo Q‐exactive MS/MS.

### 2.9 Data analysis

For the initial round of MS described in Figure 2, OIS and vector EV samples were sorted based on mean label‐free quantification (LFQ) intensities. Fold change in LFQ intensities were also calculated between conditions. Gene Ontology (GO) was then investigated with FunRich v3.1.3, using the Gene Ontology database, and cellular compartment terms ranked based upon the percentage of genes. For the second round of MS described in Figure 4, mean LFQ intensities for all proteins in each sample were used. Gene ontology was then investigated as above. Protein Atlas (www.proteinatlas.org) was then used to determine the localisation of all identified proteins and classified as either intracellular, membrane or secreted. Where localisation data indicated multiple categories, the protein was included in both lists. Finally, proteins were classified as canonical SASP components through comparison to established profiles identified in (Coppé et al., 2010). Heat Maps were then generated for each localisation category describing the mean LFQ intensity of protein for each sample.

#### 2.9.1 Enzyme‐Linked Immunosorbent Assay (ELISA)

Conditioned media was collected as per the EV isolation procedure. After the 2000 *x g* spin, 100 μl conditioned media was assessed using a commercially available Solid Phase Sandwich ELISA kit according to manufacturer's recommendations (R&D Systems, Human IL‐8 DuoSet ELISA DY208). Samples were measured at 450 and 570 nm using a CLARIOstar Plus multi‐mode plate reader (BMG Labtech). EV fractions were assessed with the same procedure without lysis.

### 2.10 Conditioned media investigations

Conditioned media was collected as per the EV isolation procedure described above. For IMR90 experiments, following the 2000 x *g* step, media was passed through a 0.22 μm sterile filter and supplemented in a 3:1 ratio with 40% FBS DMEM and 8 mM L‐glutamine in order to achieve final concentrations comparable to that of the standard media (Acosta et al., 2013). Control media was made up using serum free DMEM using the same supplement ratio. 120 μl was applied to proliferating vector cells seeded in 96‐well plates, 1 day post‐seeding and again at 4 days post‐seeding. Cells were then fixed, permeabilised and stained using the immunofluorescence protocol on day 6. For MDA‐MB‐468 treatments, 10 μl per well of conditioned media was added following the 2000 x *g* spin, to cells seeded in 96‐well plates as per the IMR90 dosing schedule. Analysis was carried out using IN Cell 2200 high content microscope and analysis system as described above.

### 2.11 Extracellular vesicle treatment investigations

Vector proliferating IMR90s or MDA‐MB‐468s were seeded in 96‐well‐plates and treated 1 day post‐seeding with 10 μl of SEC fractions or PBS vehicle control. Fraction 8 and 20 from OIS and fraction 8 from vector IMR90s were used. Only samples which had been prepared on new SEC columns were used for EV treatment experiments. Media was changed and treatment repeated on day 4, after which cells were then fixed, permeabilised and stained using the immunofluorescence protocol on day 6, with analysis carried out using IN Cell 2200 high content microscope and analysis system as described above.

### 2.12 Antibodies

For immunoblotting and immunofluorescence the following antibodies were used: p21 (12D1, Cell Signalling, UK; 1:2,000), p16 (10883‐1‐AP, Protein‐Tech, UK; 1:2,000), Ki67 (NCL‐Ki67p, Novocastra, UK;1: 1,000), IL‐8 (AF‐208‐NA, R and D Systems, UK; 1:500), CD9 (CD9A‐1, System Biosciences, UK; 1:1,000) TSG101 (ab30871, Abcam, UK; 1:1,000), Calnexin (ab22595, Abcam, UK; 1:1,000), HRP‐conjugated goat anti‐rabbit (Dako, UK; 1:5,000), HRP‐conjugated rabbit anti‐goat (Dako, UK; 1:5,000), goat anti‐rabbit‐Alexa Fluor 546 (Thermo‐Fisher, UK; 1:500), Rabbit anti‐goat‐Alexa Fluor 546 (A10040,Thermo‐Fisher, UK; 1:500).

### 2.13 Statistical analysis

Statistical analysis was performed using GraphPad Prism 7. An unpaired Student's t‐test was used to compare the means of two groups unless specified. Ordinary one‐way ANOVA followed by a Tukey's post‐hoc test was used for comparing multiple groups. *P* values represent the following: ^∗^ *P* < 0.05; ^∗∗^ *P* < 0.01; ^∗∗∗^ *P* < 0.001; ^∗∗∗∗^ *P* < 0.0001. Error bars represent SD of ≥3 independent experiments unless otherwise stated.

## 3 RESULTS

### 3.1 Isolation and proteomic analysis of EVs isolated from senescent cells using only differential ultracentrifugation

**FIGURE 1:**
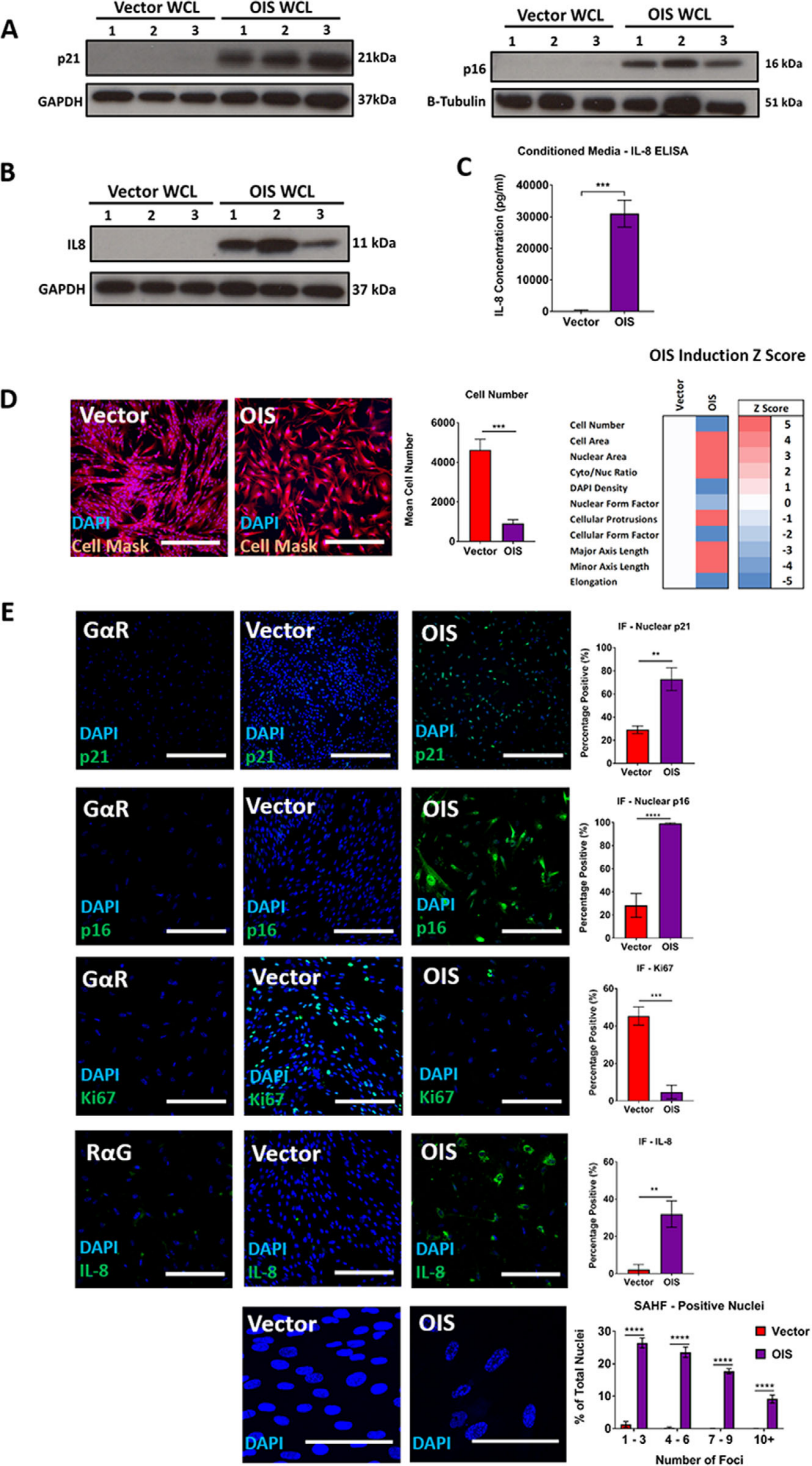
Characterising oncogene‐induced senecence phenotype through High Content Microscopy Analysis (HCA), immunoblotting and ELISA. Senescence markers were assessed by immunofluorescence and western blotting. (A) Western blot analysis of whole cell lystae (WCL) from OIS and vector cells for the cannonical senescence markers p21 and p16. Each lane is a sample from an independent experiment. *N* = 3. (B) Western blot analysis of whole cell lystae (WCL) from OIS and vector cells for the canonical SASP marker IL‐8. Each lane is a sample from an independent experiment. *N* = 3. (C) IL‐8 ELISA analysis of conditioned media from vector control and OIS cells. *N* = 3. (D) Immunofluorescence staining for DAPI (blue) and Cell Mask (Red) in OIS and vector control IMR90s. High content analysis (HCA) of cellular proliferation and morphology were quantitated. Z‐scores were calculated based upon the standard deviation in the vector (see methods). *N* = 3. (E) Immunofluorescence staining for changes in canonical senescence markers p21 (Green), p16 (Green), Ki67 (Green), IL‐8 (Green) and senescence‐associated heterochromatin foci (SAHF) (DAPI Foci) in vector and OIS cells. GαR represents secondary only control in OIS samples. *N* = 3. Scale bars: p21 = 500 μm, SAHF = 125 μm, remaining = 250 μm.

dUC is the most commonly applied technique for isolating EVs (Gardiner et al., 2016). However, it has previously been demonstrated to be limited by the co‐isolation of soluble protein contaminants (Foers et al., 2018). Despite this, it has been the most widely applied method for obtaining EVs from the culture supernatant of senescent cells (Takasugi, 2018). Here, we investigated changes in EV production in senescence through use of the well described IMR90 HRas:ER model of oncogene‐induced senescence (Serrano et al., 1997). First, we demonstrated a loss of cellular proliferation following senescence induction, accompanied by canonical changes in a panel of senescence markers (p21, p16, Ki67, IL‐8, senescence‐associated heterochromatin foci; SAHFs) (Figure 1A‐E). These conventional senescence hallmarks were used to support the development of an HCA based characterisation approach similar to Yin et al. (Yin et al., 2013), in which we sought to identify senescent cells according to a panel of established morphological characteristics. Along with the intrinsic reduction in proliferation associated with senescence, these included acquisition of enlarged and irregular cell/nuclear morphologies (Hwang et al., 2009; Sadaie et al., 2015), increased cytoplasmic to nuclear ratios (Goldstein, 1990; Neurohr et al., 2019), and reduced DAPI intensities (Roukos et al., 2015; Zhao et al., 2010). This approach represents a high‐throughput screening tool similar to the previously described usage of senescence‐associated beta‐galactosidase (Gorgoulis et al., 2019). Through this methodology, a panel of characteristic morphological changes were demonstrated to occur in OIS (Figure 1D). In order to provide support for this approach, a model of replicative senescence in primary adult HMFs was established through long‐term serial cell culture, in order to serve as a complimentary experimental system (Figure S2A). This model has been extensively validated previously within the group with a panel of senescence markers including expression of p16 and p21, increased levels of 8‐oxoguanine, reduced BrdU incorporation and increased senescence‐associated β‐galactosidase activity (Tyler et al., 2020 ‐ *preprint)*. Here, senescence induction was validated through HCA assessment and production of the SASP factor IL‐6 identified by western blotting (Figure S2B‐C). Interestingly, the SASP factor IL‐8 was not observed in the RS SASP despite its prevalence in OIS, emphasising the heterogeneous nature of SASP composition (Figure S2D).

**FIGURE 2:**
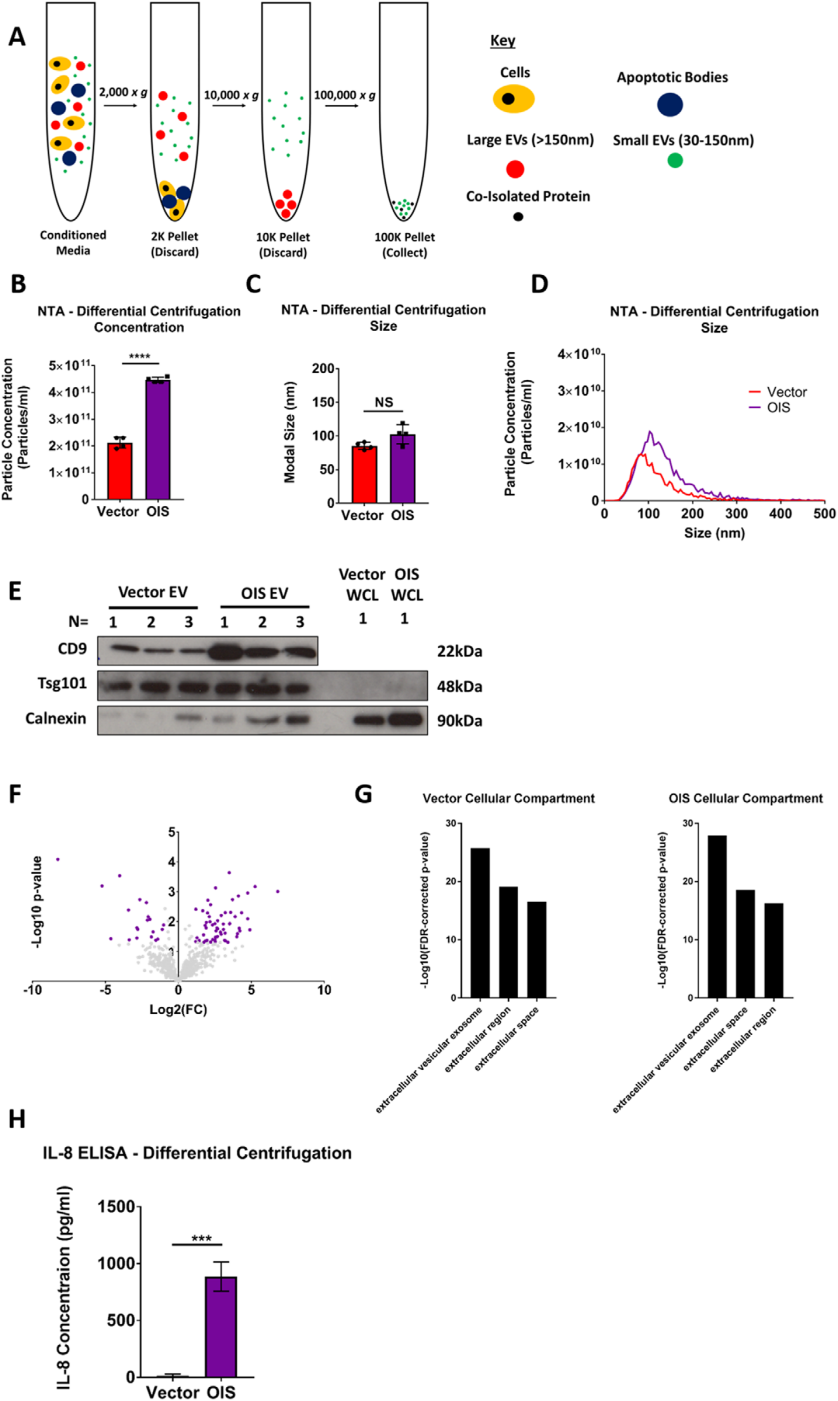
Isolation and analysis of EVs isolated from oncogene‐induced senescent (OIS) and vector proliferating control cells by differential ultracentrifugation. Extracellular vesicles (EVs) were isolated and analysed from OIS (4.3e6 cells) and vector (6.1e6 cells) control proliferating cells. (A) Workflow schematic of EV isolation methodology via differential ultracentrifugation. (B) Nanoparticle tracking analysis (NTA) particle concentration measurements of 100K pellet. *N* = 4. (C) NTA size measurements of 100K pellet. *N* = 4. (D) Full NTA size‐distribution plot. (E) Western blot analysis of EVs (*N* = 3) and whole cell lysate (*N* = 1) (WCL) from OIS and vector control cells for canonical EV markers (CD9 and Tsg101) and endoplasmic reticulum marker (Calnexin). Each lane is a sample from an independent experiment. (F) Volcano plot expressing fold change in cargo following mass spectrometry (MS) analysis between vector control and OIS cell derived EVs. *N* = 3. (G) Gene ontology analysis of 50 most abundant proteins identified by MS of EVs isolated from vector control and OIS cells. *N* = 3. (H) IL‐8 ELISA analysis of EVs from vector control and OIS cells. *N* = 3. OIS induction schedule F‐G: Vector: 8.9e6 cells, OIS: 1.7e6 cells, 48 h incubation day 7–9.

Having confirmed senescence induction, we then isolated EVs from OIS cells by dUC and analysed the 100K pellet by nanoparticle tracking analysis (NTA) (Figure 2A). An increase in EV production was demonstrated following senescence induction as compared to the proliferating control (Figure 2B). Together, these EVs had modal sizes that fell within the range typically associated with small EVs (∼100 nm) which did not vary between conditions (Figure 2C‐D). The EVs also expressed the commonly reported biogenesis marker Tsg101 and the surface marker CD9 when assessed by immunoblotting (Figure 2E). Reduced expression of the endoplasmic reticulum marker calnexin, which has been previously reported to have reduced expression in small EVs, was also observed (Figure 2E). Together, these data support previous investigations which demonstrated that EV production increases in OIS (Takasugi et al., 2017; Borghesan et al., 2019). Next, proteomic analysis was performed, assessing the composition of OIS EVs compared to those from the vector control. Differential expression was observed between conditions (Figure 2F) and GO analysis of the top 50 most abundant proteins in vector and OIS EVs suggested that, whilst EVs were enriched, soluble extracellular proteins were likely also co‐isolated (Figure 2G). Given that senescent cells have a potent secretome, it is plausible that SASP components reflect part of this co‐isolated soluble contamination. This was confirmed by ELISA, with the key SASP marker IL‐8 heavily enriched in the OIS EV samples (Figure 2H). However, dUC alone did not allow IL‐8 to be considered an EV cargo due to the issue of co‐isolating contaminating soluble protein. In order to support these observations, EVs from the RS HMFs were also isolated by dUC and assessed by NTA (Figure S3A‐C). This demonstrated an increase in EV production in late passage cells, mirroring the observations in OIS. These EVs were demonstrated to be positive for the previously reported EV marker ADAM10 (Kowal et al., 2016), although a less comprehensive assessment was performed than in the OIS samples (Figure S3D). Importantly, the EVs from RS HMFs also appeared to be associated with the SASP component IL‐6, suggesting that the limitations of dUC observed in OIS are likely recapitulated in RS (Figure S3E). Therefore, this emphasised the need for application of an isolation methodology placing more emphasis on purity, in order to elucidate the composition of senescent cell derived EVs. Because RS in HMFs requires establishment over the course of >200 days, the OIS model was selected as the more appropriate setting in which to investigate this aim, due to a greater availability of senescent cells.

### 3.2 Isolation of EVs from senescent cells by size‐exclusion chromatography allows separation of soluble and vesicular secretome

**FIGURE 3:**
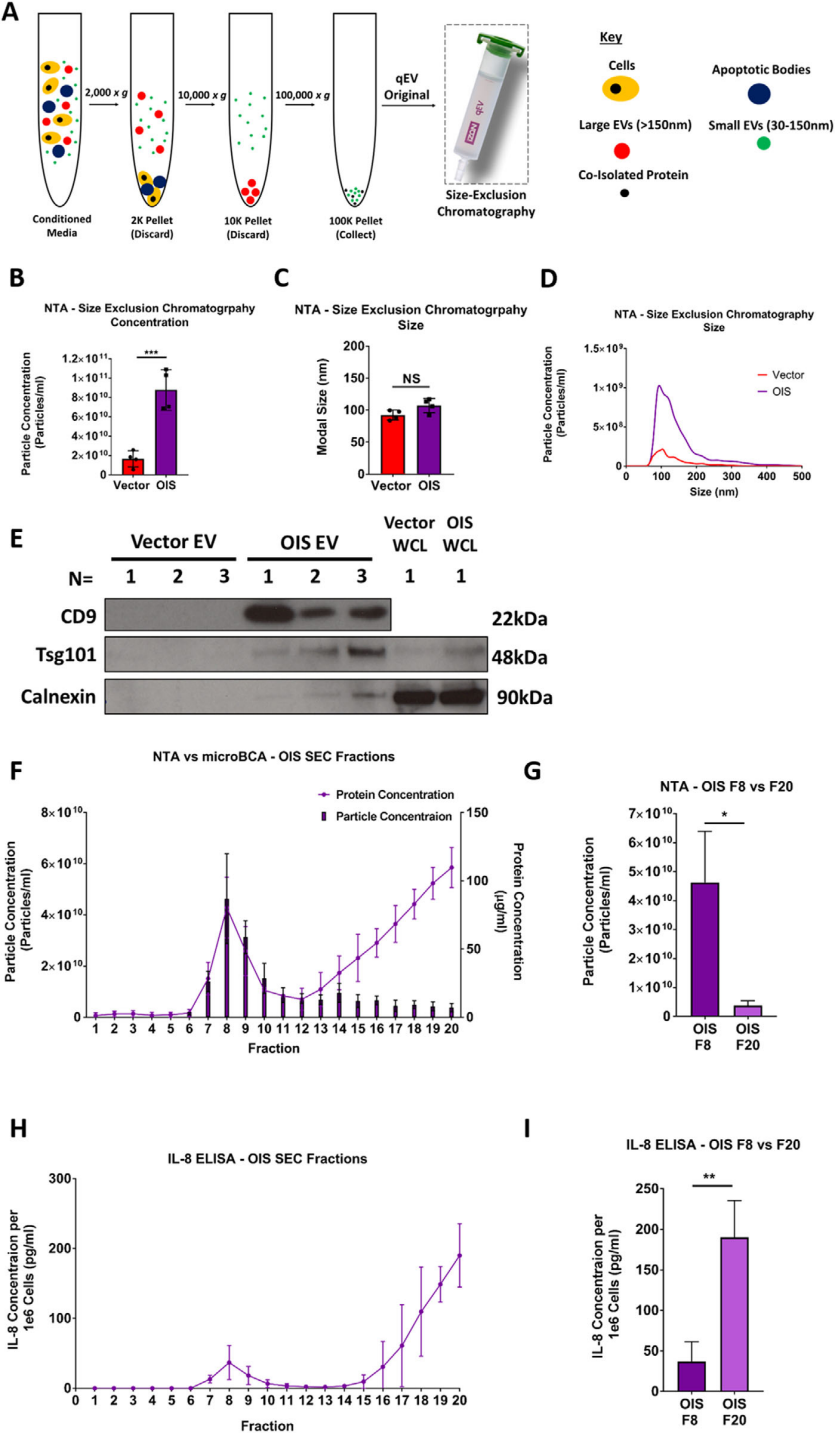
Isolation and analysis of EVs isolated from oncogene‐induced senescent (OIS) and vector proliferating control cells by size‐exclusion chromatography (SEC). Extracellular vesicles (EVs) were isolated and analysed from OIS (4.3e6 cells) and vector (6.1e6 cells) control proliferating cells. (A) Workflow schematic of EV isolation methodology via SEC. (B) Nanoparticle tracking analysis (NTA) particle concentration measurements of SEC fraction 8. *N* = 4. (C) NTA size measurements of SEC fraction 8. *N* = 4. (D) Full NTA size‐distribution plots. (E) Western blot analysis of fraction 8 EVs (*N* = 3) and whole cell lysate (*N* = 1) (WCL) from OIS and vector control cells for canonical EV markers (CD9 and Tsg101) and endoplasmic reticulum marker (Calnexin). Each lane is a sample from an independent experiment. (F) NTA particle (F6‐20) and microBCA protein (F1‐20) concentration measurements of SEC fractions from OIS cells. *N* = 3 (G) NTA particle concentration measurements for fraction 8 and 20 from OIS cells. *N* = 3. (H) IL‐8 ELISA analysis of SEC fractions (F1‐F20) from OIS Cells. *N* = 3. (I) IL‐8 ELISA analysis measurements for fraction 8 and 20 from OIS cells. *N* = 3. OIS induction schedule F‐K: Vector 5.1e7 cells, OIS: 1.6e7 cells, 72 h incubation day 8–11.

SEC was employed as an additional purification method, following EV enrichment by dUC (Figure 3A). Manufacturer instructions indicated that EVs would be enriched into fractions 7–10, whereas contaminating soluble protein, which co‐isolated during dUC, would be confined to later fractions. NTA assessment of fraction 8 EVs from OIS and vector control cells indicated that the increase in EV production following senescence induction (identified in Figure 2) was maintained following this isolation procedure (Figure 3B). Once again, these EVs had a modal size broadly consistent with that anticipated for a population of small EVs (∼100 nm) (Figure 3C‐D). However, SEC sacrifices EV yield for the sake of purity and, as such, overall concentrations were approximately only a fifth of those achieved with differential centrifugation alone. This resulted in a technical challenge to achieve sufficiently concentrated EV samples for immunoblotting, particularly in the vector condition. Despite this, as seen with the pre‐SEC samples, EVs isolated from OIS cells by SEC demonstrated enrichment for the markers Tsg101 and CD9 along with a reduced expression of calnexin, suggesting that SEC is an effective isolation methodology for enrichment of small EVs from senescent cells (Figure 3E). In order to further assess SEC, protein and particle concentrations were acquired for all 20 fractions from OIS samples. Protein concentration increased in fractions 7–10, peaking in fraction 8. From fraction 12 onwards, there was a steady increase, peaking in fraction 20 (Figure 3F). The fraction 7–10 peak was accompanied by an increase in particle concentration, again peaking in fraction 8. However, despite high protein levels, low particle concentrations were observed in the later fractions, including fraction 20 (Figure 3F‐G). This suggests that SEC allows separation of the vesicular component of the SASP from co‐isolated contaminating soluble protein. OIS fractions were then probed for IL‐8 by ELISA, in order to investigate whether this SASP factor was still associated with the EVs following SEC. Whilst some IL‐8 was detectable within the fraction 8 EVs, this analysis demonstrated that IL‐8 was predominantly associated with later fractions, indicating that this SASP factor did indeed co‐isolate with EVs via dUC (Figure 3H‐I). This data demonstrates the benefit of SEC as a means of dissecting the vesicular and soluble components of the SASP, in a way that is not achievable with dUC alone. However, NTA profiling supported immunoblotting data, which indicated low recovery yield to be a limitation of this technique, as reported previously (Figure 3K) (Coumans et al., 2017). Given this limitation, in order to provide further evidence to support the use of SEC in a senescence setting, we set about further proteomic analysis of each component of the OIS secretome.

### 3.3 Proteomic analysis of size‐exclusion chromatography fractions demonstrates that differential ultracentrifugation is insufficient to dissect soluble and vesicular SASP

**FIGURE 4:**
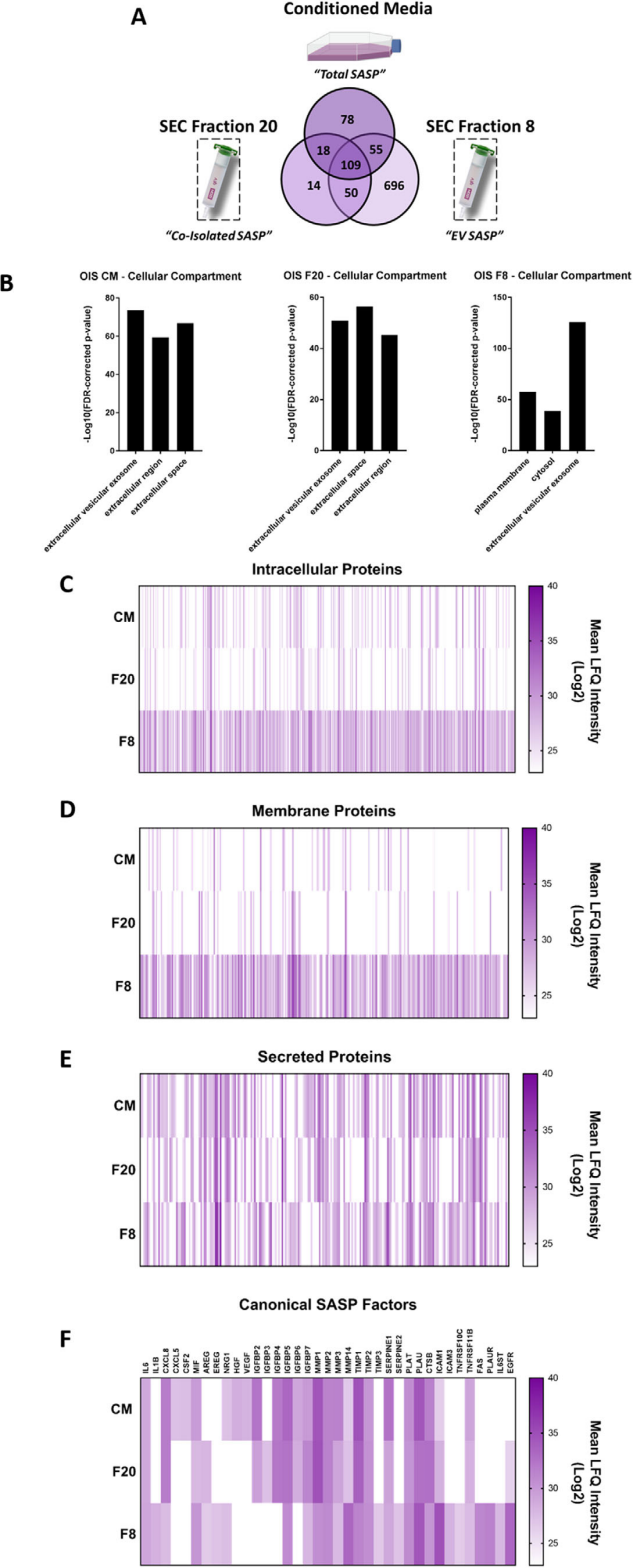
Mass Spectrometry (MS) proteomic analysis of Conditioned Media (Total SASP), SEC Fraction 20 (Co‐Isolated SASP) and SEC Fraction 8 (EV SASP) from OIS cells. (A) Venn diagrams displaying number of unique and shared proteins identified by MS between Conditioned Media (‘Total SASP’), SEC fraction 20 (‘Co‐Isolated SASP’) and SEC fraction 8 (‘EV SASP’) from OIS cells. *N* = 3. (B) Gene ontology analysis of proteins identified by MS. (C‐D) Heat Maps displaying protein localisation as either Intracellular, Membrane or Secreted and canonical SASP factors. *N* = 3. OIS induction schedule A‐F: Vector 5.1e7 cells, OIS: 1.6e7 cells, 72 h incubation day 8–11.

In order to further probe the requirement for a high purity EV isolation procedure (such as SEC) in separating the vesicular and soluble components of the SASP, proteomic analysis was performed on the conditioned media (CM; total SASP), fraction 8 (‘EV SASP’) and fraction 20 (‘co‐isolated SASP’) from OIS cells (Figure 4A). GO analysis in Figure 2 indicated that pre‐SEC EV samples also contained extracellular proteins. Post‐SEC GO analysis indicated that fraction 8 was enriched for EV proteins as well as those of the plasma membrane and cytosol (Figure 4B). By contrast, extracellular proteins were again enriched in fraction 20, although EV proteins were still represented (Figure 4B). Interestingly, the total conditioned media also appeared to have a strong EV component, further supporting the importance of this relatively underappreciated fraction of the SASP (Figure 4B). In order to support GO analysis, protein localisation was further investigated using Protein Atlas. Intracellular and membrane proteins were, again, highly enriched into fraction 8, with the majority being present only in this fraction (Figure 4C‐D). Secreted proteins, by contrast, were present across all fractions with far less enrichment (Figure 4E). This was supported by a comparable profile observed for secreted factors canonically considered components of the SASP (Figure 4F) (Coppé et al., 2010). Taken together, these data suggest that SEC is an efficient method for isolating small EVs from senescent cells and provides a means of confidently profiling the proteomic composition of senescent cell derived EVs. Furthermore, the methodology described here provides a framework that can be applied to other senescence models, which is important given the previously reported heterogeneity between SASPs (Basisty et al., 2020). Overall, this proteomic assessment highlights the need for rigor and stringency in separating the vesicular and soluble fractions of the SASP. In order to provide evidence for this requirement beyond compositional analysis, we then sought to investigate whether SEC allowed functional discrimination between these components of the senescent secretome.

### 3.4 EVs isolated from senescent cells contribute to the paracrine effects of the SASP

**FIGURE 5:**
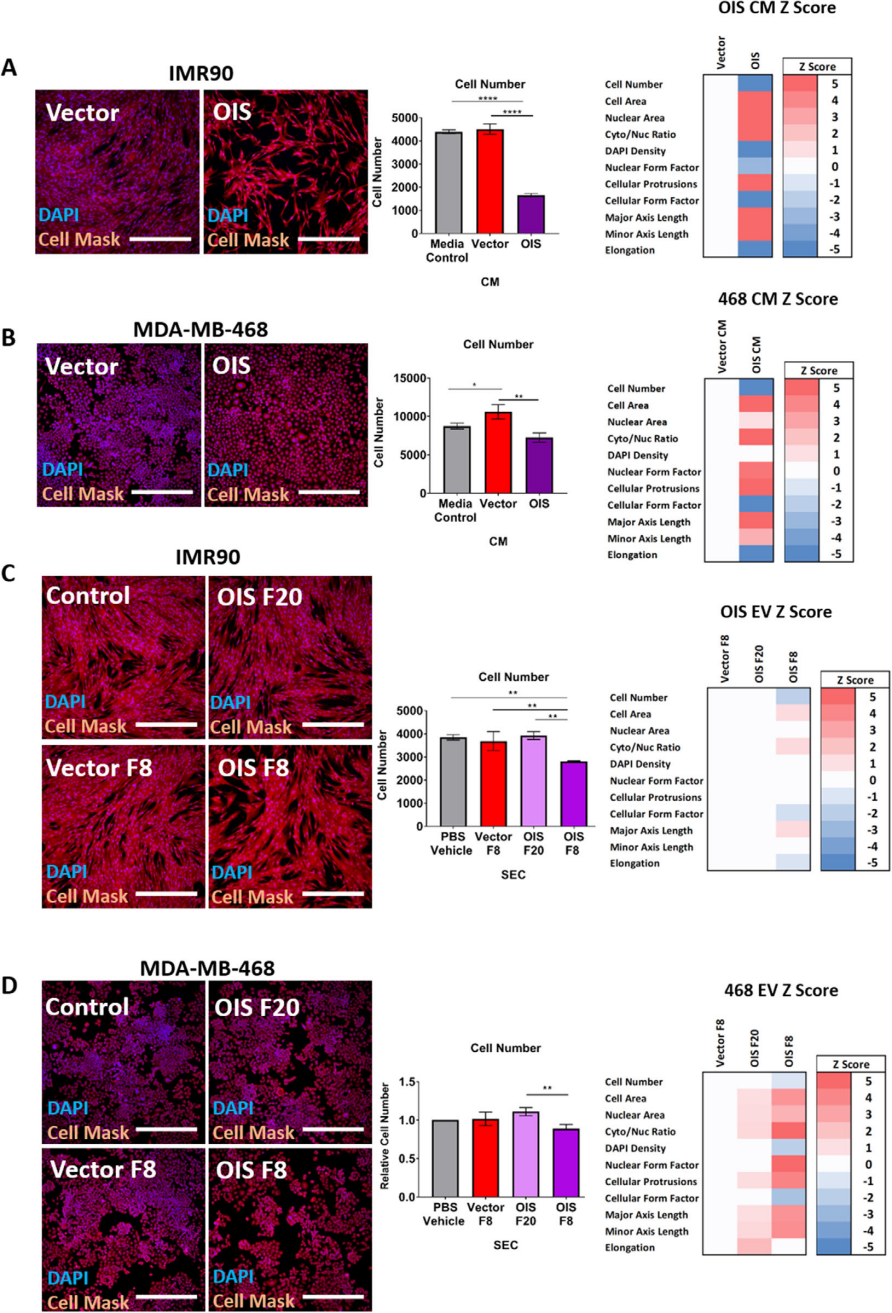
Functional Analysis of Conditioned Media and EVs to the Non‐Cell Autonomous Effect of Paracrine Senescence. (A) Proliferating fibroblasts treated with conditioned media from OIS or vector proliferating control cells. *N* = 3. (B) MDA‐MB‐468 basal like breast cancer cells treated with conditioned media from OIS or vector control cells. *N* = 3. (C) Proliferating fibroblasts treated with fraction 8 and fraction 20 from OIS and fraction 8 from vector control cells. *N* = 3. (D) MDA‐MB‐468 basal like breast cancer cells treated with fraction 8 and fraction 20 from OIS and fraction 8 from vector control cells. Relative cell numbers based upon the control per experiment are presented. Z‐scores were calculated based upon the standard deviation in the vector (see Methods). *N* = 3. Scale bars = 250 μm. Controls: CM, unconditioned media; EV, PBS vehicle.

A key role of the SASP is the ability to confer senescence upon neighbouring proliferating cells in the local microenvironment, so called paracrine senescence (Acosta et al., 2013). As described in Figure 1, we were able to unbiasedly quantitate senescence induction through morphological analysis, thus maximising the additional potential phenotypic readouts that could be generated following a single EV treatment regime. This approach demonstrated a potent paracrine senescence response through use of conditioned media from OIS cells upon both proliferating IMR90 fibroblasts and MDA‐MB‐468 basal like breast cancer cells (Figure 5A‐B) supporting previous observations (Acosta et al., 2013). In order to assess the potential contribution of EVs to this effect, fraction 8 (EVs) and fraction 20 (co‐isolated SASP) were also investigated. These were compared to a vehicle only control, as the dUC product had been comprehensively demonstrated to be an inappropriate means of EV preparation due to soluble contamination, whilst this investigation was specifically concerned with determining the utility of SEC fractions in functional experiments. In line with the results generated following treatment with vector conditioned medium (Figure 5A‐B), fraction 8 from vector control cells did not significantly alter cellular proliferation or morphology. By contrast, fraction 8 from OIS cells produced a significant reduction in cellular proliferation and morphological changes indicative of paracrine senescence in both IMR90 fibroblasts (Figure 5C) and MDA‐MB‐468s (Figure 5D). This was not recapitulated with fraction 20, indicating a distinct role for OIS EVs in mediating paracrine senescence, beyond the soluble SASP. It is important to emphasise that given the differences in EV concentrations between the vector and OIS fraction 8 samples, it is not possible to distinguish if the effect of OIS fraction 8 is due to the ‘dose’ of EVs applied or the EV cargo *per se* without further work. However, what these results illustrate is the need for methodological rigour in EV isolation, in order to elucidate the distinct functional role of EVs isolated from the secretome of senescent cells.

## 4 DISCUSSION

The biological significance of EVs as intercellular communicators is a relatively new concept. Following the demonstration that EVs could deliver functional mRNA to recipient cells, they have received widespread attention as potential novel mediators in a variety of cell types and disease settings (Valadi et al., 2007). This rapid rise to prominence resulted in a lack of standardisation in fundamental EV research methodologies, including nomenclature, isolation techniques and characterisation methods (Gould & Raposo, 2013; Lötvall et al., 2014; Tkach & Théry, 2016). This was compounded by the technical challenges of EV research, which stem primarily from their small size and the lack of universal markers (Van Niel et al., 2018). Given this, efforts by the International Society for Extracellular Vesicles (ISEV) to achieve rigour and standardisation are ongoing and have made progress in harmonising the field under the MISEV guidelines (Lötvall et al., 2014; Théry et al., 2018).

Mirroring the EV field, senescence research is also hindered by a lack of universal markers, as well as heterogeneity between cell types and senescence triggers (Gorgoulis et al., 2019). This is particularly true of the SASP, which also varies in composition throughout the course of senescence induction and between specific cellular contexts (Hoare et al., 2016; Basisty et al., 2020). Therefore, there are significant technical challenges in marrying these two emerging fields and elucidating the composition and functions of senescent cell derived EVs. Applying stringent methodologies is key to overcoming these challenges, as well as laying the foundation for studies focussing on functional cargos and mechanisms. Here, we demonstrate that SASP factors co‐isolate with EVs through dUC, making it an ineffective tool when applied alone. SEC allows separation of the vesicular and soluble composition of the SASP, thus making it a more suitable technique for determining the makeup and functional role of EVs within senescence. This has facilitated generation of a comprehensive profile of OIS EVs, which could provide a useful resource for the selection of potential functional targets in future investigations. However, as described above, senescence reflects a diverse set of phenomena and it would be prudent to follow up this work in additional senescence models, such as RS HMFs, as the specific profiles generated here must be considered OIS specific. Nevertheless, we hope that the experimental approaches described may provide a blueprint for such future profiling of senescence cell derived EVs in other settings.

Furthermore, we have demonstrated that the previously described effect of paracrine senescence can be replicated following use of SEC in two models, providing proof‐of‐principle that SEC can be used to determine the distinct role of EVs within the SASP, complimenting previous studies (Borghesan et al., 2019). This could be supported in future work, particularly in mechanistic investigations, by application of additional senescence markers, in order to more comprehensively describe these effects. However, due to the context specific nature of these so‐called ‘hallmarks’, this work made use of a high‐throughput screening tool of senescence induction, with the aim of establishing the principle that SEC does not preclude investigation of EVs in paracrine senescence investigations (Gorgoulis et al., 2019).

As the fields of EV and senescence research develop and continue to converge, we believe the heterogeneity within sub‐populations of EVs (Kowal et al., 2016) and different senescent cell contexts (Gorgoulis et al., 2019) will add nuance to their potential role within the SASP (Wallis et al., 2020). In order to explore this complexity, it is crucial that senescence researchers appreciate the recent advances within the EV field and apply the same level of rigor and stringency laid out in the MISEV guidelines (Lötvall et al., 2014; Théry et al., 2018). Recent publications suggest that awareness of these considerations may be growing (Borghesan et al., 2019; Alibhai et al., 2020; Mensà et al., 2020). However, given the historical nescience within the field, more needs to be done to widen understanding of this key issue (Choi, Kil, & Cho, 2020; Riquelme et al., 2020). Therefore, we hope this study will highlight the importance of selecting appropriate methodologies when conducting EV research, particularly in the senescence field, where the enhanced secretome of the soluble SASP has the potential to be a particularly potent contaminant.

## CONFLICT OF INTERESTS

The authors declare no competing interests.

## Supporting information

Figure S1

Figure S2

Figure S3

Table S1

Table S2
