# Supplementary figures and images for: Isolation methodology is essential to the evaluation of the extracellular vesicle component of the senescence‐associated secretory phenotype
Source: J Extracell Vesicles. 2021 Feb 18;10(4):e12041. doi: 10.1002/jev2.12041 (PMC7892802; doi:10.1002/jev2.12041)

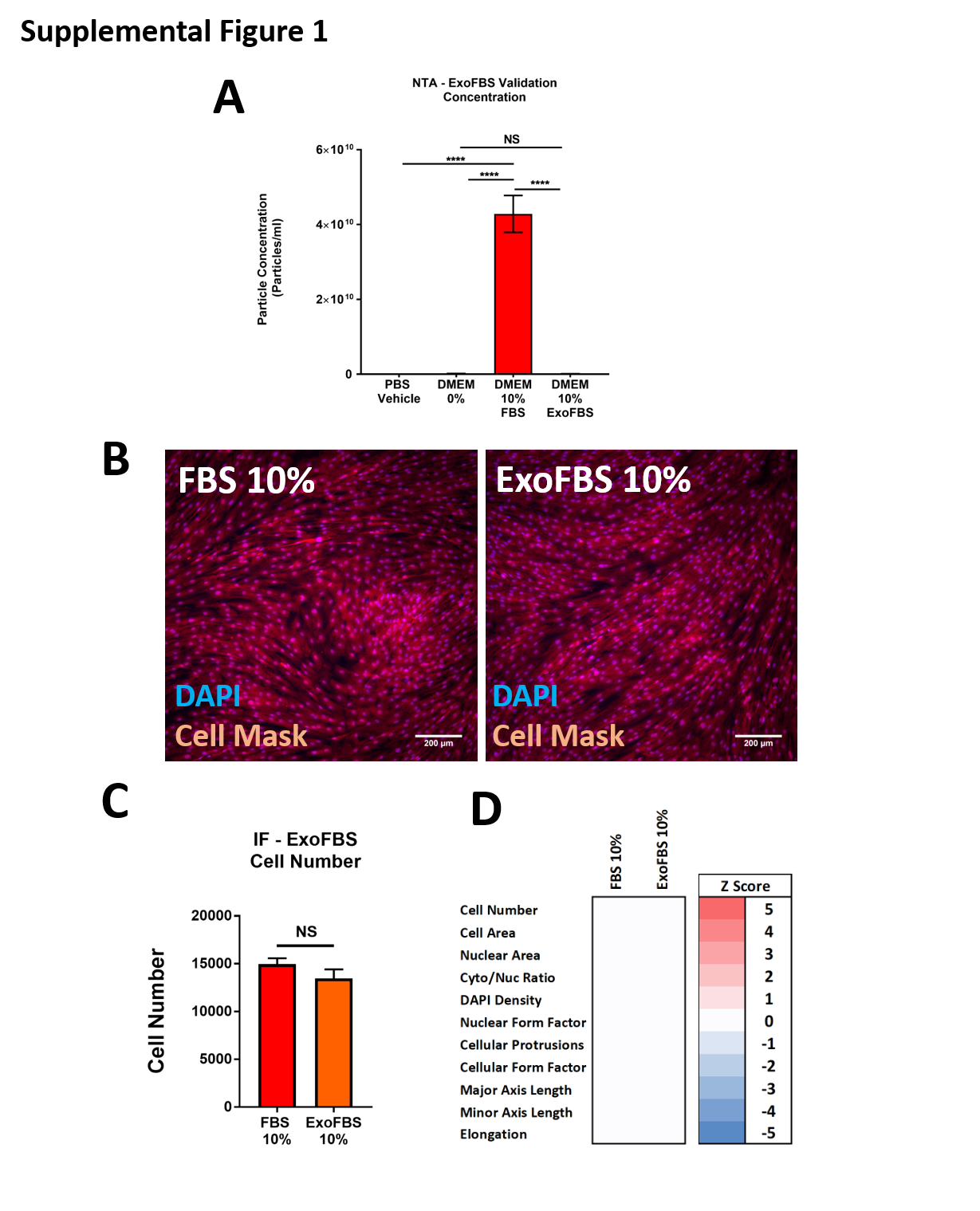

Supplement: Supplementary file 1 — Figure S1 [file JEV2-10-e12041-s001.tif]

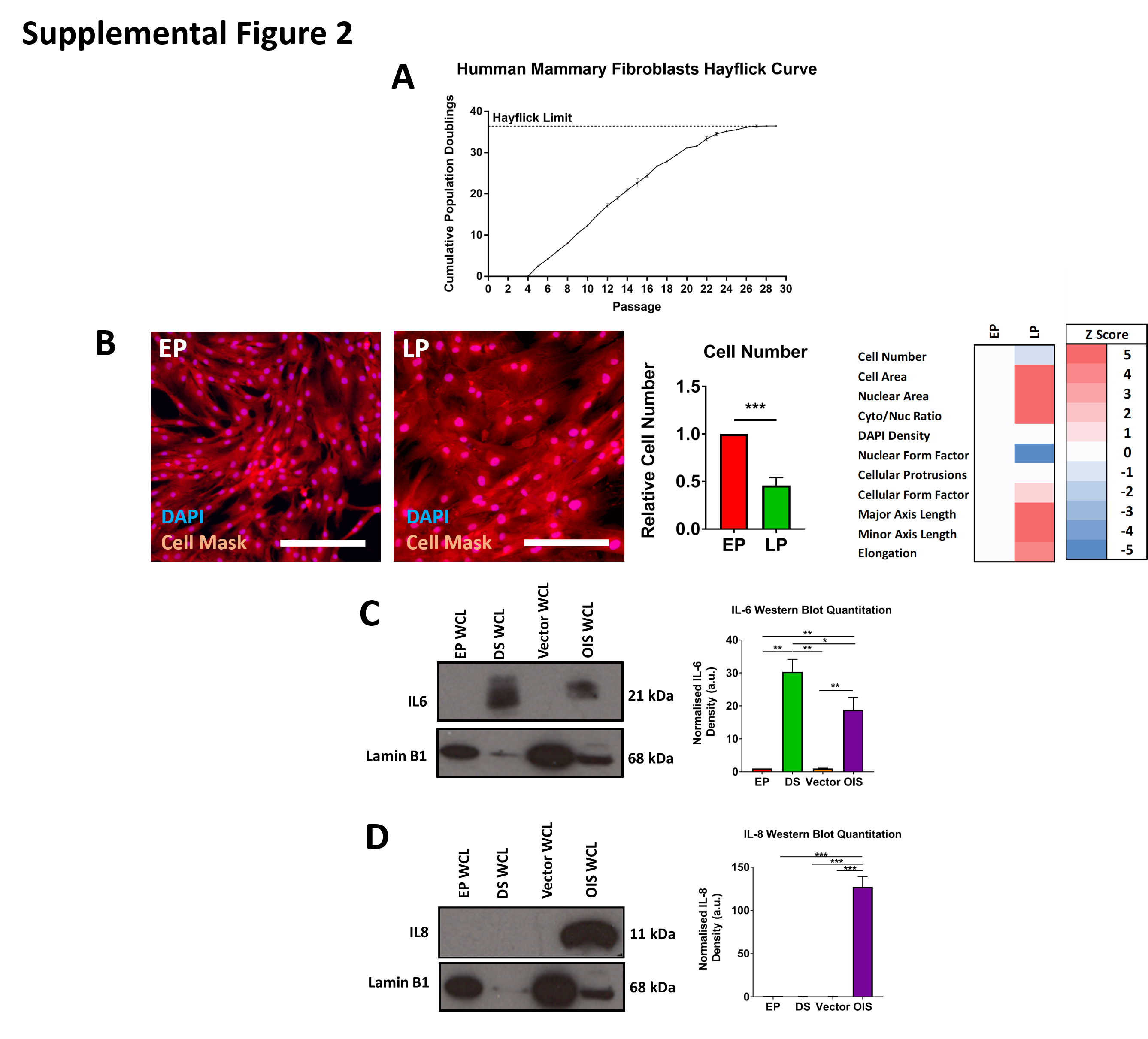

Supplement: Supplementary file 2 — Figure S2 [file JEV2-10-e12041-s002.tif]

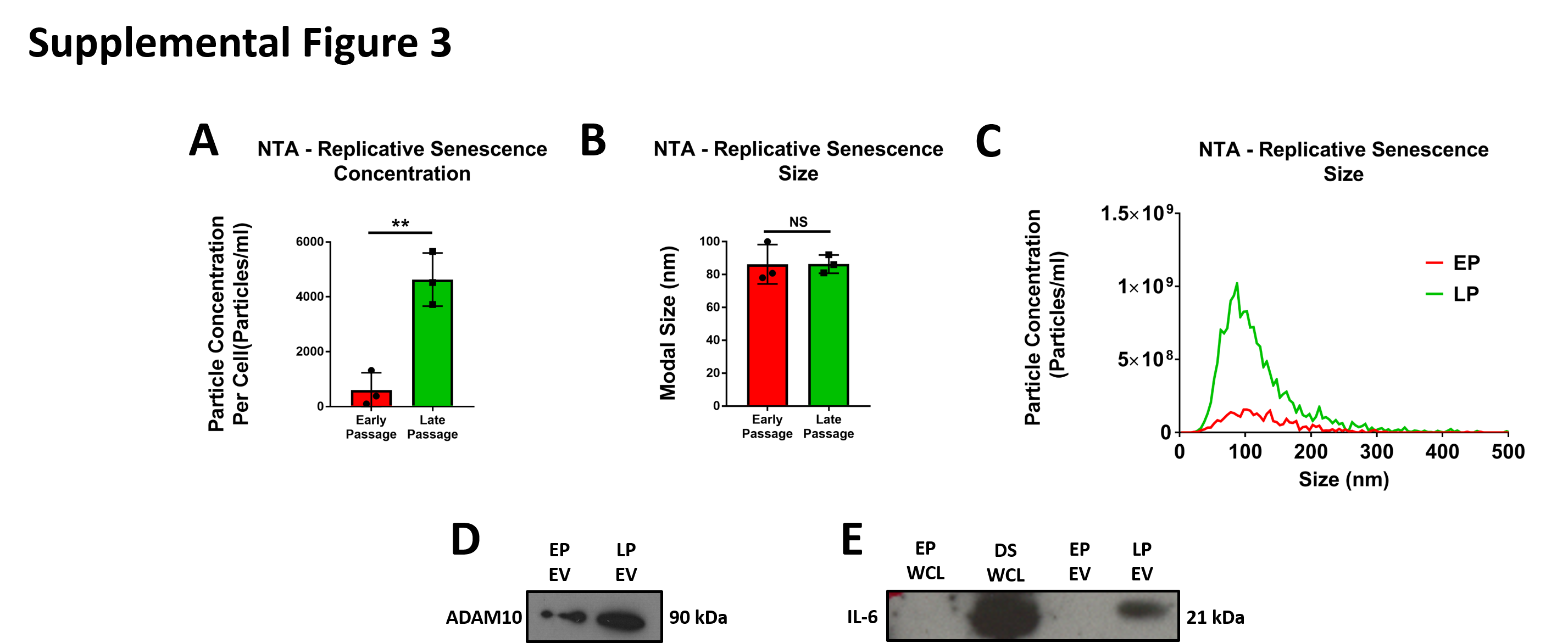

Supplement: Supplementary file 3 — Figure S3 [file JEV2-10-e12041-s003.tif]
